# Quantifying the AI readiness gap: An international, multidisciplinary assessment of artificial intelligence literacy in the radiation oncology community

**DOI:** 10.1016/j.ctro.2026.101210

**Published:** 2026-06-01

**Authors:** Ciaran Malone, Dylan Callens, Elizabeth Forde, Michelle Leech, Carlos Cardenas, Mark J. Gooding, Samantha Ryan, Pierre Thirion, Claire Fitzpatrick, Theresa O’Donovan, Antony Carver, Irene Hernandez Giron, Darragh P. Browne, Catherine Rogerson, Brendan McClean, B.J.M. Heijmen, Gerard G. Hanna, Jill Nicholson

**Affiliations:** a Erasmus MC Cancer Institute, University Medical Center Rotterdam, Department of Radiotherapy, the Netherlands; b St. Luke’s Radiation Oncology Network, St. Luke’s Hospital, Dublin, Ireland; c Laboratory of Experimental Radiotherapy, KU Leuven, Leuven, Belgium; d Department of Radiation Oncology, University Hospitals Leuven, Leuven, Belgium; e Trinity St. James’s Cancer Institute, Trinity College Dublin, Dublin, Ireland; f Applied Radiation Therapy Trinity, Discipline of Radiation Therapy, Trinity College Dublin, Ireland; g Department of Radiation Oncology, University of Alabama at Birmingham, Birmingham, AL, USA; h Inpictura Ltd, Abingdon, UK; i Division of Cancer Sciences, Faculty of Biology, Medicine and Health, The University of Manchester, Manchester, UK; j Discipline of Medical Imaging and Radiation Therapy, School of Medicine, University College Cork, Ireland; k Department of Medical Physics, University Hospitals Birmingham NHS Foundation Trust, Birmingham, UK; l Centre for Physics in Health and Medicine, School of Physics, UCD, Dublin, Ireland

**Keywords:** Artificial intelligence, Radiation oncology, Clinical competence, Medical education, Psychometrics, Health workforce

## Abstract

**Purpose:**

The rapid integration of artificial intelligence (AI) into imaging-intensive fields like radiation oncology (RO) is transforming the clinical workforce from manual operators to supervisory validators, yet the baseline competencies required for safe oversight remain undefined. Here we present the first consensus-built, validated international knowledge assessment of AI literacy to quantify the RO knowledge profile.

**Methods:**

A multidisciplinary, international panel from clinical practice, academia, and industry developed a 22-item knowledge assessment mapped to core AI-in-radiotherapy concepts. Items underwent a three-round consensus process with blinded initial voting, achieving > 80% panel agreement. The instrument was deployed worldwide via a multilingual web application, and demonstrated excellent internal consistency (Cronbach’s α = 0.90) and strong discrimination (mean: 0.63).

**Results:**

760 individuals engaged with the assessment; 528 completed all items. Significant role-specific disparities emerged: Medical Physicists demonstrated higher scores than Radiation Oncologists and Radiation Therapists (p < 0.001). However, required competencies vary by role. While the workforce exhibited strong intuition regarding data-centric risks such as bias and data drift, widespread deficits were identified in fundamental model mechanics and interpretation of prediction uncertainty. This differential performance hinders the shared vocabulary necessary to describe model failures or distinguish between simpler models and advanced foundational architectures capable of adapting to complex clinical data, both labelled “AI”. Our analysis also challenges traditional educational paradigms: targeted workshops proved as effective as formal degrees, and knowledge score did not correlate with career stage.

**Conclusions:**

These results establish a global benchmark for AI readiness. While clinical staff may not require AI development expertise, our findings demonstrate significant value in targeted training to build shared AI vocabulary, ensuring clinical adoption does not outpace safe oversight.

## Highlights

• First validated AI literacy tool for the radiation oncology workforce (n = 528).
• AI literacy varies significantly by role, with medical physicists scoring highest.
• Workforce excel in data-centric AI concepts but lack model mechanic literacy.
• Workshops are as effective as formal degrees for rapid AI upskilling.
• AI knowledge does not correlate with career stage or clinical experience.

## Graphical abstract

**Figure:**
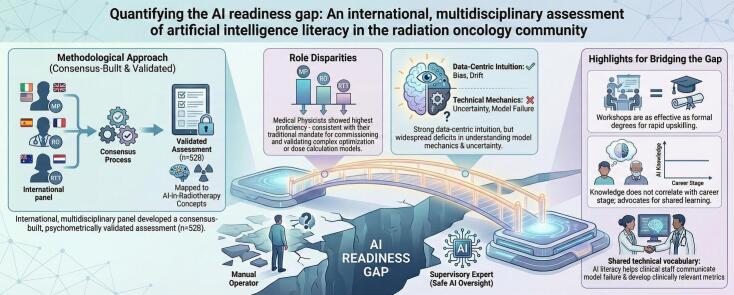

## Introduction

The discipline of radiation oncology is currently navigating a technological transition of unprecedented magnitude.[1], [2], [3] Historically, the field has been defined by both software and hardware innovation.[4] However, the current revolution towards Artificial Intelligence (AI) integrated radiation oncology is fundamentally different. AI is a foundational technology that reshapes not only the workflow[5], [6] but the cognitive architecture [1], [7] of oncology practice, transitioning the radiation oncology workforce from a paradigm of manual creation to one of supervisory validation[1], [2]. However, effective supervision requires what Azad et al. describe as 'visible guardrails'.[8] Without an understanding of model mechanics, the decision-making logic remains opaque to the end-user.[9] This shift necessitates a fundamental re-evaluation of what constitutes clinical competence. In AI image segmentation, competent staff now 'audit' pre-generated structures rather than manually contouring slice-by-slice. This requires a higher order of anatomical knowledge and a sophisticated understanding of how the algorithm might fail.[10], [11] Reviewing an AI-generated contour now involves consideration whether a deviation is a subtle anatomical variant induced by an “out-of-training-domain” sample or “data-drift”, concepts critical to effective oversight.[12], [13].

This technological acceleration has highlighted a critical “readiness gap”. [7], [13], [14], [15] While the deployment of sophisticated AI based tools appears to have outpaced the educational infrastructure necessary to support their safe and effective use, the actual extent of the disparity remains largely anecdotal and unquantified. Despite the rapid adoption of these tools, it is currently unknown whether the workforce possesses the baseline competencies required for safe oversight. A growing body of evidence points to an “enablement gap” where high enthusiasm for, and awareness of, AI is matched by low self-reported competence.[2], [16], [17], [18], [19], [20].

To bridge the gap between rapid AI development and workforce skills, professional societies in Radiation Oncology have begun to mobilize to define ethical principles[21], regulatory position papers[22], and technical guidelines for model development/deployment[23], [24], [25], [26], [27]. Specific clinical guidance is also emerging, such as recommendations on auto-contouring[28] and frameworks for Large Language Models[27]. Yet, a disconnect remains: while high-level guidelines are proliferating, formalised AI curricula for the wider clinical workforce remain absent or in early development stages. Currently, competencies are explicitly mandated only within Medical Physics core curricula and training programmes[29], [30], [31], leaving the broader clinical team without structured preparation despite the clear need for targeted education.[32], [33], [34], [35].

The primary objective of this initiative was to quantify the current AI-related knowledge profile and oversight skills across professional groups, which may help professional societies and educators identify areas of weaker performance to benchmark curricula, target and prioritise training needs, and evaluate interventions across the Radiation Oncology community. Secondary objectives were to characterise item performance, evaluate reliability of the instrument, and explore the relationship between AI knowledge and self-reported experience, attitudes, and training.

## Methods

### Instrument development and content mapping

An international, multidisciplinary panel with 9 members was convened, including Radiation Oncologists (ROs), Medical Physicists (MPs), Radiation Therapists (RTTs) and industry/academic experts, with representation from ESTRO AI-focus group. Using a consensus-based item generation method, members proposed and reviewed potential questions to ensure coverage of essential AI-in-radiotherapy concepts, generating an initial question bank of 55 single-best-answer multiple choice questions. These concepts included: basic terminology and task types; data, labels and performance metrics; model generalisability and dataset shift; bias and fairness; clinical oversight and governance; model training/evaluation terminology; and typical limitations and failure modes of AI systems used in radiotherapy workflows. Items were written to target two main user roles: (i) AI users, focusing on recognition of correct terminology and basic concepts, and (ii) AI monitors/evaluators, focusing on limitations, weaknesses and biases required for clinical oversight. All items were formatted as single-best-answer multiple-choice questions (MCQs) designed to be answerable by a typical Radiation Oncology professional without programming background. Distractor options were constructed to be plausible for the target audience and to reflect common misconceptions where possible.

### Multidisciplinary consensus process

Content refinement followed a three-round consensus process. For each question and round, the panel indicated “accept as is”, “revise” or “reject” using a simple voting system implemented on a per-question basis, and they could add free-text comments and suggested rephrasings. In Round 1, individual votes and comments were not visible to other panel members to minimise anchoring and dominance effects. In Round 2, voting was repeated and all voting and comments were visible, ensuring a transparent multidisciplinary consensus. Items with < 50% consensus between the 9 members were removed. Items with mixed or high “revise” votes were iteratively rewritten to address concerns about ambiguity, distractor quality, relevance or difficulty. Round 3 focused on resolving remaining disagreements. Items achieving > 80%[36] consensus were retained, ensuring each targeted an essential AI principle, used clear terminology, and had one unambiguous correct answer with realistic distractors. Items that could not be satisfactorily resolved were removed. Remaining items were consolidated into a final set of 22 knowledge items (Table S1) with the panel ensuring coverage of a range of important concepts, terminology and common misconceptions from data-centric AI to technical model mechanics.

### Demographic items

Alongside the knowledge questions, we developed non-identifiable targeted demographics. These included: professional discipline, career stage, prior AI-related training, self-rated AI knowledge, self-rated comfort in using AI-related terminology, main role/responsibilities and perceived importance of AI related knowledge.

### Recruitment and eligibility

The assessment was implemented in Python using the Streamlit framework to create a responsive custom web-based application in 7 languages (Dutch, Spanish, French, Italian, German and Danish) (See supplementary material for app details). The app was accessible globally via a public URL and hosted on Streamlit between 1st September 2025 and 30th November 2025. Participation was voluntary and open to individuals working or training in Radiation Oncology, including ROs, MPs and RTTs. The survey link was disseminated via ESTRO National Societies, professional email lists, local institutional mailing lists, and social media channels targeting the Radiation Oncology community. Ethical Approval for the study was granted by the local research ethics committee (REC: NR/2025/1).

### Scoring and outcome measures

Each knowledge item was scored as 1 for a correct answer and 0 for any incorrect choice. For each respondent, a total knowledge score was calculated as the sum of correct responses across the 22 items and expressed as a percentage. Item-level performance was summarised as the proportion of respondents selecting the correct answer among those who responded to that item. Categorical variables derived from demographic items were used to describe the cohort and to examine associations between respondent characteristics and knowledge scores. Primary analysis included only respondents who completed all 22 knowledge items.

### Psychometric validation and statistical analysis

Internal consistency of the knowledge scale (Q8-Q29) was assessed with Cronbach’s α[37]. Item discrimination was evaluated using the Kelley 27% discrimination index (difference in proportion correct between the top and bottom 27% of total scores)[38]. Item discrimination was interpreted with common convention, treating values around or above 0.30 as acceptable for an applied educational instrument[39]. Knowledge score was the percent correct across Q8-Q29, restricted to respondents who completed all 22 items. For multi-level factors (profession, AI training, self-rated knowledge, self-rated comfort, career stage, perceived importance), the Kruskal-Wallis omnibus tests and two-sample Mann-Whitney U post-hoc comparisons with Holm adjustment was used within each factor to correct for multiple comparisons. All reported p values are Holm corrected unless stated otherwise. To validate statistical robustness, a parallel independent Bayesian analysis was undertaken using a Student-t likelihood model with weakly informative priors centered on the global mean (σ_prior = 2σ_observed). The 95% Highest Density Interval (HDI) was calculated for pairwise differences; differences were considered credible if the HDI excluded zero.[40] This dual-method approach allowed robust effects to be distinguished from those sensitive to conservative multiple-testing penalties.

### Item specific performance

While overall question performance was reported as a baseline, question items were further categorized into two broad thematic groups (e.g. Understanding of model training mechanics and terminology vs. understanding the impact of clinical Data/QA on model performance) and stratified by discipline. This approach allowed for the identification of distinct priority learning needs, role-specific strengths, specifically aiming to distinguish between deficits in technical computational knowledge and areas where existing clinical expertise facilitates intuitive understanding of AI concepts.

### Sensitivity analysis

For completeness, to assess whether dropout biased the primary findings, sensitivity analyses were conducted on the full cohort of all initiated sessions for RO, MP and RTTs (n = 685). This included per-item differences between completers and non-completers, and re-testing of each demographic factor. Full details of the testing methodology & results can be found in Supplementary Material.

## Results

### Participation and respondent characteristics

**Fig. 1:**
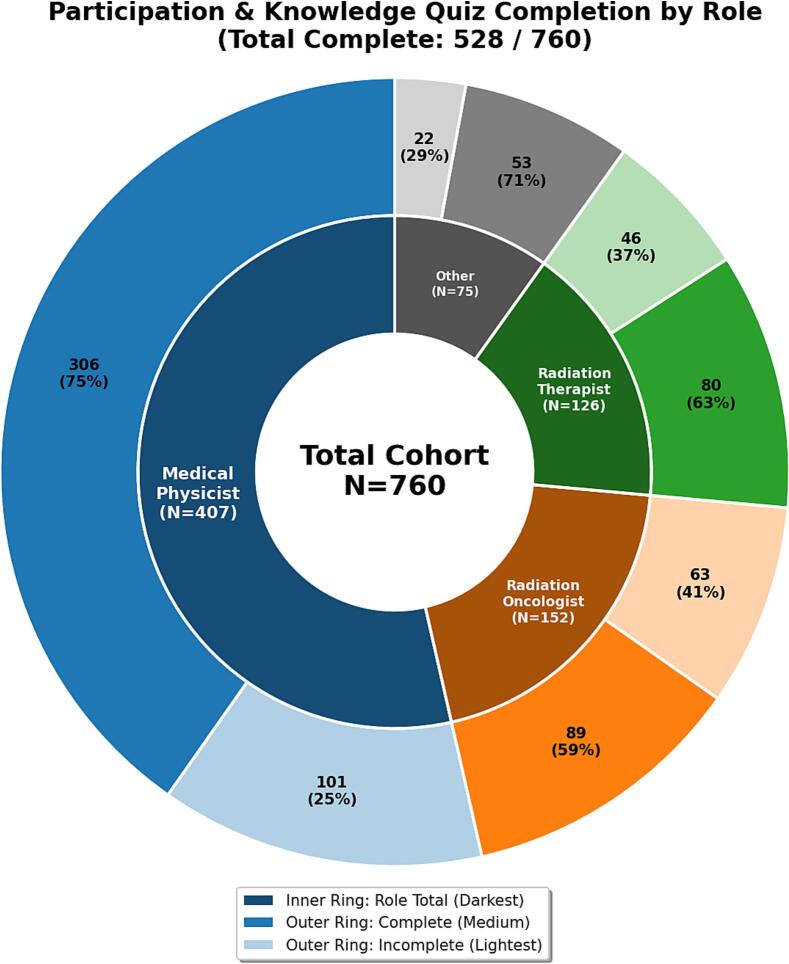
Participation and completion rates by professional role. Nested donut chart displaying the total participant cohort (n = 760). Inner ring represents total participants per role, while the outer ring represents quiz completion status (darker = completed all knowledge questions, lighter = incomplete knowledge questions (Q8-Q29)). ROs had the highest incomplete rate (41%), followed by RTTs (37%) and MPs (25%). “Other” includes Engineers, Quality Managers, Industry professionals, Nursing, Researchers/Academics and Software Engineers.

A total of 760 individuals initiated the knowledge assessment; 528 completed all 22 items, corresponding to a completion rate of ∼ 70%. For reference, question/answer pairs reported in Table S1. The cohort included 306 MPs, 89 ROs, 80 RTTs, and 53 respondents who selected “Other” professional roles (Fig. 1). While formal geographic data was not captured, 68 respondents (∼13%) used one of five non-English languages, indicating multi-regional reach. Only those who completed all knowledge items are reported on in this analysis.

### Psychometric validation results

The 22-item knowledge scale demonstrated high internal consistency, with Cronbach’s α = 0.90. Item discrimination was good to very good, with a mean discrimination index of 0.63 ± 0.13 (range 0.32–0.82). No items demonstrated negative or negligible discrimination (Table S2).

### Knowledge performance

**Fig. 2:**
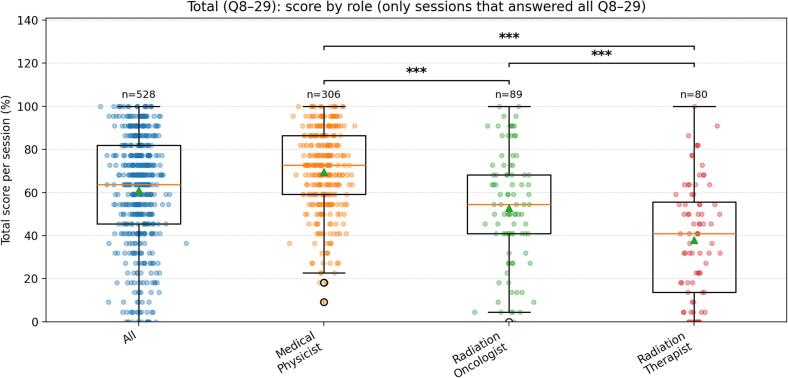
Distribution of total knowledge scores across the workforce. Box and jitter plots showing percentage scores for all participants who completed all knowledge based questions (Q8-29) (n = 528) and stratified by professional role. The box represents the interquartile range (IQR), the central line indicates the median. Medical Physicists (MPs; n = 306) scored significantly higher than Radiation Oncologists (ROs; n = 89) and Radiation Therapists (RTTs; n = 80). Green triangles represent the mean score for each group. (For interpretation of the references to colour in this figure legend, the reader is referred to the web version of this article.)

The cohort’s median score was 63.6% (interquartile range [IQR] 45.5–81.8), with significant professional disparities (p < 0.001) (Fig. 2). MPs achieved the highest scores, with a median of 72.7% [59.1–86.4] (n = 306). ROs had a median score of 54.5% [40.9–68.2] (n = 89), and RTTs had a median of 40.9% [13.6–55.7] (n = 80). Pairwise comparisons showed all between-group differences were statistically significant after Holm correction (all p < 0.001). Independent Bayesian analysis confirmed these findings (Fig. S1,Table S3).

### AI training, self-rated knowledge and comfort

**Fig. 3:**
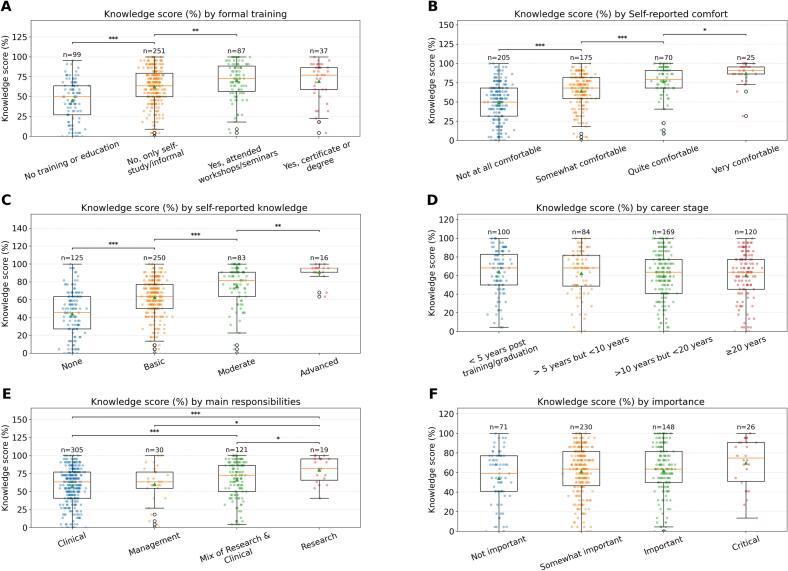
Factors associated with AI knowledge performance across Medical Physicists (MPs), Radiation Oncologists (ROs), and Radiation Therapists (RTTs). Box plots displaying knowledge scores stratified by: (A) level of formal AI training; (B) self-reported comfort with AI terminology; (C) self-rated AI knowledge level; (D) career stage (years post-qualification); (E) primary professional responsibility; and (F) perceived importance of AI in Radiation Oncology. Centre lines show medians; box limits indicate the 25th and 75th percentiles. Green triangles represent group means. (For interpretation of the references to colour in this figure legend, the reader is referred to the web version of this article.)

Level of prior AI training was strongly associated with performance (Fig. 3, p < 0.001). Median scores increased with increasing levels of training. Any form of training, including informal self-study, yielded significantly higher scores compared to no training (p < 0.001). Workshops/seminars provided a significant performance boost vs. self-directed learning (p = 0.013) (Fig. 3A). Formal certificates/degrees also showed no advantage over self-study after Holm correction (p = 0.09), confirmed by independent Bayesian analysis to be not significant. Scores did not differ between workshops/seminar participants and formal certificates/degrees holders (p = 0.95). Self-rated AI knowledge and self-rated comfort both showed strong positive associations with objective scores (p < 0.001; Fig. 3B-C, Table 1). However, a subset of respondents who self-rated as “None” or “Basic” achieved scores in the upper range of the cohort.

**Table 1:** Analysis of Medical Physicist, Radiation Oncologist and Radiation therapist respondent characteristics associated with AI knowledge scores. Comparisons of total knowledge scores across professional roles, career stage, prior training, self-perception, and attitudes. Note: Global differences were assessed using the Kruskal-Wallis test (Reporting H, with degrees of freedom in parentheses). Pairwise comparisons were performed using Mann-Whitney U tests with Holm correction to correct for multiple comparisons (p_holm). n1 and n2 denote sample sizes for the respective groups in pairwise comparisons. The Bayesian column reports an independent probabilistic check; “Significant” indicates that the 95% Highest Density Interval (HDI) of the difference excludes zero (full model details available in Supplementary Material). “*” denotes a difference between Bayesian & Frequentist methods.

**Factor**	**Type**	**Group 1**	**Group 2**	**n1**	**n2**	**H (df)**	**p_raw**	**p_holm**	**Bayesian**
Professional Role	Kruskal-Wallis	All	All			93.9 (2)	<0.001		
	Pairwise	Medical Physicist	Radiation Therapist	306	80		<0.001	<0.001	Significant
	Pairwise	Medical Physicist	Radiation Oncologist	306	89		<0.001	<0.001	Significant
	Pairwise	Radiation Oncologist	Radiation Therapist	89	80		<0.001	<0.001	Significant
Career Stage	Kruskal-Wallis	All	All			2.6 (3)	0.46		
	Pairwise	< 5 years post training/graduation	> 5 years but < 10 years	100	84		0.80	0.80	−
	Pairwise	< 5 years post training/graduation	>10 years but < 20 years	100	169		0.16	0.98	−
	Pairwise	< 5 years post training/graduation	≥20 years	100	120		0.33	1	−
	Pairwise	> 5 years but < 10 years	>10 years but < 20 years	84	169		0.27	1	−
	Pairwise	> 5 years but < 10 years	≥20 years	84	120		0.43	1	−
	Pairwise	>10 years but < 20 years	≥20 years	169	120		0.68	1	−
Comfort Level	Kruskal-Wallis	All	All			111.4 (3)	<0.001		
	Pairwise	Not at all comfortable	Quite comfortable	205	70		<0.001	<0.001	Significant
	Pairwise	Not at all comfortable	Very comfortable	205	25		<0.001	<0.001	Significant
	Pairwise	Not at all comfortable	Somewhat comfortable	205	175		<0.001	<0.001	Significant
	Pairwise	Somewhat comfortable	Very comfortable	175	25		<0.001	<0.001	Significant
	Pairwise	Somewhat comfortable	Quite comfortable	175	70		<0.001	<0.001	Significant
	Pairwise	Quite comfortable	Very comfortable	70	25		0.01	0.01	Significant
Formal Training	Kruskal-Wallis	All	All			53.8 (3)	<0.001		
	Pairwise	No training or education	Yes, attended workshops/seminars	99	87		<0.001	<0.001	Significant
	Pairwise	No training or education	No, only self-study/informal	99	251		<0.001	<0.001	Significant
	Pairwise	No training or education	Yes, certificate or degree	99	37		<0.001	<0.001	Significant
	Pairwise	No, only self-study/informal	Yes, attended workshops/seminars	251	87		0.00	0.01	Significant
	Pairwise	No, only self-study/informal	Yes, certificate or degree	251	37		0.04	0.09	−
	Pairwise	Yes, attended workshops/seminars	Yes, certificate or degree	87	37		0.96	0.96	−
Importance Level	Kruskal-Wallis	All	All			7.7 (3)	0.05		
	Pairwise	Not important	Critical	71	26		0.01	0.07	Significant*
	Pairwise	Not important	Somewhat important	71	230		0.07	0.21	−
	Pairwise	Important	Critical	148	26		0.12	0.25	−
	Pairwise	Not important	Important	71	148		0.07	0.27	−
	Pairwise	Somewhat important	Critical	230	26		0.06	0.32	−
	Pairwise	Somewhat important	Important	230	148		0.78	0.78	−
Knowledge Level	Kruskal-Wallis	All	All			107.8 (3)	<0.001		
	Pairwise	None	Moderate	125	83		<0.001	<0.001	Significant
	Pairwise	None	Basic	125	250		<0.001	<0.001	Significant
	Pairwise	None	Advanced	125	16		<0.001	<0.001	Significant
	Pairwise	Basic	Advanced	250	16		<0.001	<0.001	Significant
	Pairwise	Basic	Moderate	250	83		<0.001	<0.001	Significant
	Pairwise	Moderate	Advanced	83	16		<0.001	<0.001	Significant
Responsibility	Kruskal-Wallis	All	All			23.9 (3)	<0.001		
	Pairwise	Clinical	Research	305	19		<0.001	<0.01	Significant
	Pairwise	Clinical	Mix of Research & Clinical	305	121		<0.01	<0.01	Significant
	Pairwise	Management	Research	30	19		0.01	0.04	Significant
	Pairwise	Mix of Research & Clinical	Research	121	19		0.07	0.08	Significant*
	Pairwise	Management	Mix of Research & Clinical	30	121		0.23	0.45	−
	Pairwise	Clinical	Management	305	30		0.49	0.49	−

### Career stage, responsibility and perceived importance of AI

Career stage was not associated with knowledge scores (Fig. 3D, p > 0.80). Role responsibilities highlighted that those involved with research scored significantly higher than those in clinical or management roles (p < 0.01)(Fig. 3E). Perceived importance of AI for the future of Radiation Oncology was mixed. Overall, 36.7% of respondents rated AI as “important” or “critical”, 48.4% as “somewhat important”, and 14.9% as “not important” (Fig. 3F). Notably, independent Bayesian analysis identified credible differences in two knowledge score comparisons (‘Not Important’ vs ‘Critical’; ‘Research’ vs ‘Mix’) where frequentist significance was observed but subsequently lost after Holm correction (Table 1, Fig. S1).

### Item specific performance

**Fig. 4:**
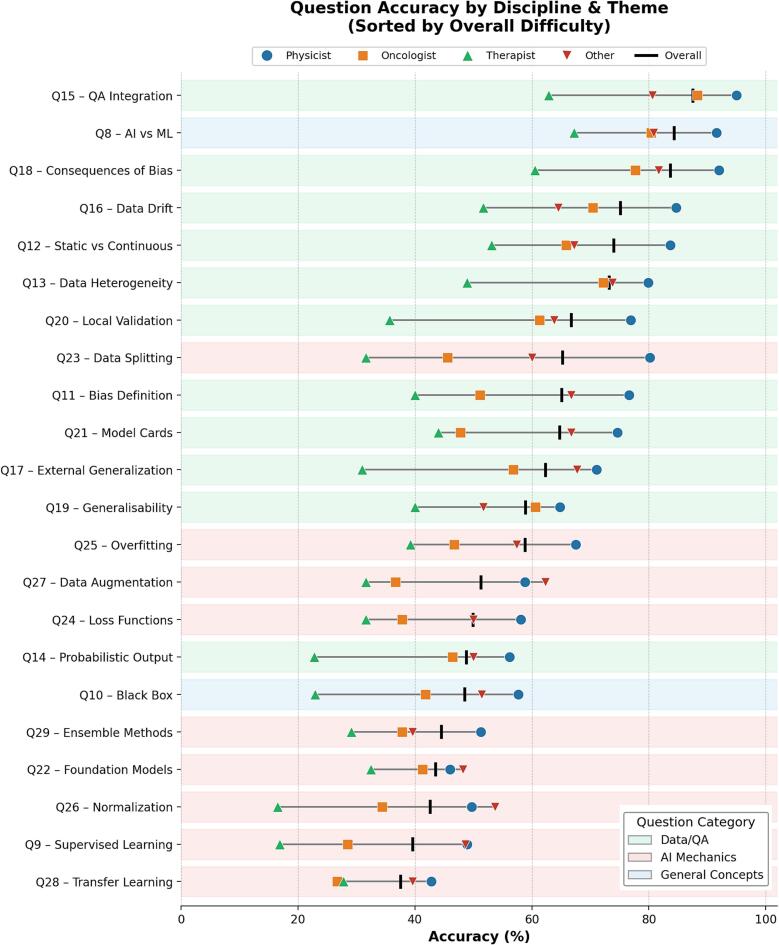
Analysis of knowledge scores per discipline and overall. Questions are thematically stratified to emphasize the distinction between technical model mechanic leaning questions (Red band) and questions related to real-world clinical data and its impact on model performance (Green band). This grouping highlights a clear area of weaker performance: low-performing questions clustered heavily around the mechanics of model training (e.g., normalization, transfer learning, loss functions), while high-performing topics centred on clinical integration, governance, and safety (e.g., QA, bias, data drift). The Blue band represents general terminology that doesn't fit into either Green or Red categories. (For interpretation of the references to colour in this figure legend, the reader is referred to the web version of this article.)

Question scores varied substantially both between questions and across disciplines (question/answer pairs reported in Table S1). Overall, participants typically scored poorly on model training mechanics (e.g., supervised learning, foundational models, transfer learning, ensembles, normalisation and loss functions), while excelling in data-centric considerations (e.g., bias, healthcare data, and QA concepts) (Fig. 4). Distinct role-based profiles emerged when stratifying performance. MPs uniquely excelled at validation methodology (such as data splitting) yet struggled with emerging architecture terminology like “foundation models”. ROs and RTTs scored best in clinical data context questions around AI, such as the clinical practice changes (data drift), data quality/heterogeneity and bias in real world data. Despite these strengths, ROs and RTTs scored low on optimization concepts/terminology (such as normalisation, data augmentation and loss functions). Notably, the fundamental concept of “supervised learning” scored poorly across all disciplines, despite its centrality to medical imaging AI. Also, both RTTs and ROs scored poorly on interpreting the certainty of model output predictions, with the majority selecting options indicating deterministic certainty rather than probabilistic likelihood of an AI predictive output.

### Sensitivity & bias analysis

Sensitivity analyses on the full cohort of all initiated sessions (n = 685 across MP, RO and RTT) confirmed that the role finding (MP > RO > RTT), the training-effect finding (no significant workshop-vs-degree difference in any cohort), and the null career-stage finding (no significant pairwise difference in any of 24 comparisons across four cohort definitions) are robust to dropout. Per-item analysis identified one question out of all 22 (Q8 (AI vs ML), the first and most-completed scored item) where Medical Physicist completers outperformed non-completers by 11 percentage points (p_holm_ = 0.025); no other role item comparison reached significance (Full details in Supplementary Material).

## Discussion

This study presents the first consensus-built, validated instrument designed to quantify the Artificial Intelligence knowledge within the international radiation oncology (RO) workforce. Supported by a robust cohort (n > 500), and excellent reliability metrics (alpha = 0.90) and strong item discrimination (0.63 ± 0.13), the instrument provides an empirical baseline of current knowledge against which professional bodies, educators, and individual departments can define their own role-appropriate standards. The results reveal that while some foundational concepts are generally understood, other deeper mechanistic knowledge and terminology is lacking, and proficiency varies significantly across professional roles. Crucially, the data challenges two paradigms of traditional training. First, the lack of correlation between knowledge and career stage questions the standard “apprenticeship” model. [41] Second, the comparable performance of respondents with formal degrees versus those attending workshops validates the critical role of agile, short-form education in this rapidly evolving domain.

### Professional disparities and clinical safety

Our knowledge assessment reveals a contrast of knowledge between technical AI model training literacy and clinical intuition of how data might influence model performance. Distinct profiles of MPs, ROs and RTTs highlight specific potential vulnerabilities in the current AI multidisciplinary workflow.

MPs achieved the highest proficiency, a finding consistent with their traditional mandate for technology commissioning, quality assurance (QA), and validating complex optimization or dose calculation models. ROs and RTTs demonstrated strong intuition for “data-centric” issues (e.g., data heterogeneity, data drift and bias), intuitively understanding that “Big Data” in medicine is rarely clean or uniform. However, despite ROs and RTTs providing the “ground truth” contours that drive AI model training, they scored poorly on the definition of supervision in an AI training context. Similarly, a deficit was observed across all disciplines regarding optimization concepts (e.g., loss functions, data augmentation choices) poses a barrier to effective model commissioning. While clinical staff do not need to engineer these functions, foundational AI literacy is essential to: 1) look beyond “AI” marketing to distinguish simple, potentially “brittle” models from advanced foundational architectures that may perform better across diverse data; and 2) facilitate communication that ensures model development decisions align with real-world clinical relevance. Also, without some foundational knowledge clinical staff may fail to articulate or recognise erroneous model behaviour, increasing the risk that subtle but clinically significant errors will go undetected.[1], [8], [42], [43] For example, the data indicates a tendency to view AI predictions as absolute certainties rather than probabilistic thresholds. This misunderstanding creates a high risk of “automation bias” (over-trust)[1], where a plausible but incorrect prediction may be accepted because they overestimate the system's deterministic nature. This is particularly concerning given recent warnings that we "cannot solely depend on the human-in-the-loop to detect problems" when such bias exists, and that safety emerges not from flawless model performance but from "knowing when not to act" on a model output.[8] AI literacy must augment, not replace, clinical judgment. Critical appraisal skills remain core, and the risk of over-reliance grows when literacy displaces rather than supports clinical reasoning.

Currently, formal AI competencies are concentrated in the Medical Physics discipline[28], [30], [43], mirroring our findings that MPs scored relatively well. However, equivalent frameworks for ROs and RTTs remain absent. RTTs serve as the frontline “human-in-the-loop” at the treatment console, while ROs bear ultimate clinical responsibility, making appropriate training across disciplines vital for safe AI model deployment. This stands in stark contrast to Clinical Radiology, where the EU/UK curricula has explicitly required trainees to appraise or understand the basic principles of AI based tools.[44], [45] The rapid formalisation of AI competencies in our sister discipline suggests that Radiation Oncology risks falling behind in preparing its clinical workforce for the same digital ecosystem. The drivers of these role-based disparities likely extend beyond curriculum mandates alone, plausibly reflecting differential AI workflow exposure (MPs commissioning systems, RTTs operating them, ROs reviewing outputs), variation in professional society emphasis, and CPD time constraints. Disentangling these motivational and structural factors will require dedicated investigation

### Agility of workshops vs. formal degrees

An important finding for educational policymakers is the efficacy of short-form training, with both self-study & workshops/seminars resulting in significantly higher scores vs. no training. Notably, respondents who engaged in workshops or seminars performed comparably with those holding formal degrees or certificates in AI. This suggests that given the rapid pace of AI evolution, agile educational formats may be more efficient for maintaining competency than static, traditional single or multi-year educational structures. However, workshops and seminars are typically voluntary in nature and not yet integrated into mandated curricula or CPD requirements. With 53% of all respondents reporting self-study, it suggests there is significant interest in upskilling AI skills (Fig. 3A). However, formal curricula may still have a critical role to play ensuring a standardised baseline of knowledge for the future workforce. Successful precedents for a formalised yet modular model are already emerging, offering a template for the broader medical community.[46], [47], [48], [49], [50], [51] However, to the authors knowledge no major professional body has yet mandated AI education for ROs or RTTs.

### A case for shared learning

The most culturally significant finding is the lack of association between knowledge and career stage where the rapid integration of technology means educators may be as adept at using new tools than the learners they supervise.[41], [52] In this assessment, a senior staff member with 20 years of experience is statistically no more knowledgeable than an early career staff member. Consequently, the traditional top-down mentorship model may be insufficient. Departments should embrace shared learning environments where early career stage staff (who may have more exposure to modern computational concepts) and senior staff (who typically possess deep clinical judgment) learn to evaluate AI tools together. Also, given that radiology often serves as the upstream input for radiotherapy workflows, collaborative training with radiology colleagues who may already be navigating established AI curricula, could provide a practical accelerator for radiation oncology teams struggling to bridge knowledge gaps.

### Self-perception of AI knowledge

The strong correlation between self-rated comfort and objective scores validates the use of self-assessment as a scalable proxy for workforce monitoring. However, the subset achieving high scores despite low self-rated knowledge reveals a “hidden talent” pool, potentially reflecting intimidation by unfamiliar AI terminology rather than true knowledge deficits. Educational interventions should therefore aim to empower staff, demonstrating how existing analytical and data skills transfer directly to AI oversight.

## Strengths & limitations

Strengths include the sample size (n = 528) and the application of dual statistical framework: a Bayesian analysis confirmed the robustness of findings, effectively separating true effects from those penalized by the frequentist approach's conservative Holm correction. Regarding instrument design, questions were developed via multidisciplinary consensus to target specific known misconceptions. However, phrasing could theoretically influence item performance. As with any expert-derived instrument, item content reflects panel priorities of what knowledge is important in Radiation Oncology today. The assessment’s difficulty and length likely contributed to the ∼ 30% drop-out rate, potentially introducing selection bias favouring respondents with higher self-perceived competence. Sensitivity analyses on the full 685-respondent cohort confirmed that the primary role, and key demographic findings were preserved across all dropout-inclusion thresholds tested. Any small differences between completers and non-completers favoured completers, suggesting that completer-only estimates may modestly overstate workforce knowledge and that the reported areas of weaker performance are, if anything, conservative. Respondents likely also represent an AI-engaged subset of each profession; findings should therefore be interpreted as the current knowledge profile of an interested workforce rather than a random sample. It is also possible users participated multiple times which due to the anonymised nature of the test, we could not detect. The fixed item pool also presents a challenge for longitudinal monitoring, as future score improvements could reflect memory retention of specific question/answers rather than true gains in AI competency. Finally, the concepts tested reflect the current AI landscape and will require periodic updates as technology evolves.

## Conclusion

This study provides a validated measure of AI literacy in radiation oncology, revealing significant role-specific knowledge profiles. While the workforce is generally adapting well to data-driven concepts around AI performance, participants scored poorest on technical terminology and model mechanic concepts potentially requiring targeted education. Crucially, such literacy empowers staff to look beyond generic “AI” labelling to distinguish simple architectures from more advanced foundational models which may possess very different performance characteristics. Addressing areas of weaker performance either through education for staff, or local AI experts facilitates effective multidisciplinary communication and ensures that the promise of AI does not outpace our ability to oversee it safely. The comparable efficacy of workshops and formal degrees supports agile, modular training for rapid workforce development, while the absence of any career-stage effect supports shared learning models. These findings provide an empirical foundation for professional societies designing AI curricula, departmental educators planning CPD, researchers studying clinical AI adoption, and individual practitioners benchmarking their own knowledge, moving the conversation about workforce AI readiness from anecdote to evidence.

## AI disclosure

During the preparation of this work Gemini was used on the final draft to help with reduction of word count, grammar and clarity throughout. At all times Gemini was asked to provide “targeted suggestions”, not overall bulk re-writes. Responses were checked for hallucinations/errors.

The author(s) reviewed and edited the content as needed and take(s) full responsibility for the content of the publication.

## Funding statement

Research Fellowship grant SLICR 2023/7542 (Applies to: Ciaran Malone, Jill Nicholson, Samantha Ryan).

## CRediT authorship contribution statement

**Ciaran Malone:** Conceptualization, Data curation, Formal analysis, Investigation, Methodology, Project administration, Validation, Visualization, Writing – original draft, Writing – review & editing. **Dylan Callens:** Conceptualization, Investigation, Methodology, Validation, Writing – original draft, Writing – review & editing. **Elizabeth Forde:** Conceptualization, Investigation, Methodology, Validation, Visualization, Writing – original draft, Writing – review & editing. **Michelle Leech:** Investigation, Methodology, Validation, Visualization, Writing – original draft, Writing – review & editing. **Carlos Cardenas:** Conceptualization, Investigation, Methodology, Validation, Visualization, Writing – original draft, Writing – review & editing. **Mark J. Gooding:** Conceptualization, Investigation, Methodology, Validation, Visualization, Writing – original draft, Writing – review & editing. **Samantha Ryan:** Conceptualization, Investigation, Methodology, Validation, Visualization, Writing – original draft, Writing – review & editing. **Pierre Thirion:** Conceptualization, Investigation, Methodology, Validation, Visualization, Writing – original draft, Writing – review & editing. **Claire Fitzpatrick:** Investigation, Methodology, Validation, Visualization, Writing – original draft, Writing – review & editing. **Theresa O’Donovan:** Conceptualization, Data curation, Investigation, Methodology, Validation, Visualization, Writing – original draft, Writing – review & editing. **Antony Carver:** Investigation, Methodology, Validation, Visualization, Writing – original draft, Writing – review & editing. **Irene Hernandez Giron:** Methodology, Validation, Visualization, Writing – original draft, Writing – review & editing. **Darragh P. Browne:** Methodology, Validation, Writing – original draft, Writing – review & editing. **Catherine Rogerson:** Writing – original draft, Writing – review & editing. **Brendan McClean:** Methodology, Supervision, Writing – original draft, Writing – review & editing. **B.J.M. Heijmen:** Supervision, Validation, Visualization, Writing – original draft, Writing – review & editing. **Gerard G. Hanna:** Conceptualization, Data curation, Investigation, Methodology, Supervision, Validation, Visualization, Writing – original draft, Writing – review & editing. **Jill Nicholson:** Conceptualization, Data curation, Formal analysis, Investigation, Methodology, Project administration, Supervision, Validation, Visualization, Writing – original draft, Writing – review & editing.

## Declaration of Competing Interest

The authors declare the following financial interests/personal relationships which may be considered as potential competing interests: Ciaran Malone, Samantha Ryan and Jill Nicholson reports financial support was provided by St.Lukes Institute of Cancer Research (SLICR) which is a charitable body. If there are other authors, they declare that they have no known competing financial interests or personal relationships that could have appeared to influence the work reported in this paper.

## Appendix A Supplementary data

Supplementary Data 1 Detailed web-application methodology and translation procedures, the complete AI knowledge survey instrument and response options, comprehensive Bayesian pairwise analyses of demographic score differences, and sensitivity analyses evaluating cohort dropout bias.
