# Supplementary material for: Quantifying the AI readiness gap: An international, multidisciplinary assessment of artificial intelligence literacy in the radiation oncology community
Source: Clin Transl Radiat Oncol. 2026 Jun 1;59:101210. doi: 10.1016/j.ctro.2026.101210 (PMC13251754; doi:10.1016/j.ctro.2026.101210)
Supplement: Supplementary Data 1 — Detailed web-application methodology and translation procedures, the complete AI knowledge survey instrument and response options, comprehensive Bayesian pairwise analyses of demographic score differences, and sensitivity analyses evaluating cohort dropout bias. [file mmc1.docx]

Supplementary Material

## Supplementary Material A: Web-app additional information:

**App implementation and data management**
The assessment was implemented in Python using the Streamlit framework to create a responsive custom web-based application. The app presented a brief introduction and information statement, followed by the demographic items, and the 22 knowledge questions. Responses were captured client-side and transmitted securely to a Google Cloud backend using an authenticated service account. Each respondent's session data were written in real time to a dedicated Google Sheets workbook, with each row corresponding to a unique response and each column representing a single question item answer or metadata field. No identifiable personal information was collected. The link between session identifiers and responses allowed quality checks for duplicate submissions and incomplete records.

**Translation**
The English version was treated as the reference instrument. The 22 knowledge items and all demographic and perception items were translated into six additional languages (Dutch, Spanish, French, Italian, German and Danish) by ChatGPT (v.5). Each language had a spot-check by a bilingual Radiation Oncology professional. A clear disclaimer was added to the quiz to highlight English as the reference language, and ChatGPT was used to translate other language options.

## Supplementary Material B: Full item bank and response options

Table S1. **Full item bank and response options.** List of the Seven questions regarding participant details and 22 knowledge-based questions and demographic items used in the assessment, including all distractor options. Correct answers for knowledge items are indicated. Questions presented to each participant.

| ID | Question | Response Options |
| --- | --- | --- |
| 1 | Which of the following best describes your professional role? | 1. Radiation Oncologist  2. Medical Physicist  3. Radiation Therapist  4. Other (Dosimetrist, Researcher, etc.) |
| 2 | What stage are you in your career? | 1. < 5 years post training/graduation  2. > 5 years but <10 years  3. >10 years but <20 years  4. >20 years |
| 3 | What best describes your main role/responsibilities? | 1. Research  2. Clinical  3. Management  4. Mix of Research & Clinical |
| 4 | Have you received any formal education or training in AI or machine learning? | 1. Yes, as part of a degree/course  2. Yes, attended workshops/seminars  3. No, only self-study/informal  4. No training or education |
| 5 | How would you rate your current level of knowledge of the strengths and limitations of AI-based tools used in clinical practice? Pick the highest level where most statements fit you. | 1. No knowledge: Unfamiliar with key AI terms (e.g., U-Net, LLM, sensitivity/specificity, generalisability, bias); little or no practical use of AI tools.  2. Basic: Know common terms at a high level; can use approved tools with guidance; not comfortable with model cards or validating models.  3. Moderate: Confident with common terms; can read/use model cards; understand typical strengths/limits; can run local validation.  4. Advanced: Lead or advise on validation/QA and monitoring (drift, bias, calibration), set/justify thresholds, align use with governance/regulation, teach or mentor others. |
| 6 | How comfortable are you with accurately using AI-related terminology? (e.g. UNET vs. LLMs, Generative AI, AI vs machine learning, foundational models AI hallucinations)? | 1. Not at all comfortable  2. Somewhat comfortable  3. Quite Comfortable  4. Very comfortable |
| 7 | How important is it for your current job role to understand AI concepts? | 1. Not at all  2. Somewhat important  3. Very important  4. Critical |
| 8 | What is a fundamental distinction between artificial intelligence (AI) and machine learning (ML)? | 1. AI and machine learning are interchangeable terms describing any advanced software capable of learning from big data.  2. AI refers only to rule-based expert systems, while machine learning describes algorithms that make predictions without using any prior data  3. AI is a broad field encompassing systems that perform tasks requiring human-like intelligence, while machine learning is a subset of AI focused on building models that learn patterns from data  4. Machine learning is used for automating administrative tasks, while AI is only used for complex image analysis |
| 9 | What is Supervised Learning in the context of AI models? | 1. A model that is validated by a human after training is complete  2. The AI system must be supervised by a human during every use  3. A model that checks and verifies that input data matches its original training data  4. AI model that was trained with paired input and output labels |
| 10 | What does the term "black box" AI model mean? | 1. Models whose internal decision-making processes are not easily interpretable/accessible by users  2. AI software that runs on computers without monitors/visual output  3. AI models whose internal decision making can only be accessed by the vendor  4. Once trained, a models internal decision-making process cannot be accessed. |
| 11 | In relation to AI applications "model bias" most accurately refers to: | 1. The AI model only accepting inputs of a particular demographic/disease type  2. An institution's personal preference for certain AI models due to superior performance on local data.  3. Systematic errors in AI outputs resulting from non-representative or flawed training data  4. The AI model systematically changing over time due to its use on a non-diverse setting or local group of patients. |
| 12 | What distinguishes a "continuously learning" AI model from traditional "static" AI models in radiotherapy? | 1. Adaptive models are designed to be trained multiple times, while static models can only be trained once before clinical deployment.  2. Models that only work with manual “seed” input from a human which the model uses as a starting point  3. Static models cannot be used to solve problems they have never seen before, while adaptive models can be used to solve problems not seen by the model during training.  4. Adaptive models are designed to incorporate new data and improve performance over time while in clinical use |
| 13 | Which statement is true regarding healthcare data used for AI model training? | 1. Healthcare data has variability in data formats, acquisition protocols, and inconsistent labelling across institutions  2. With enough data, differences in healthcare data from similar clinical departments averaged out  3. Due to clinical consensus guidelines, healthcare data usually has uniform structure and clearly defined labels  4. Healthcare data can be considered consistent if acquired from the same clinical department |
| 14 | For a well trained and appropriately validated AI model, if the model classifies a tumour as cancerous, what is the most appropriate interpretation? | 1. The model is certain the tumour is malignant because it output a positive label.  2. The models reported accuracy during validation was 90%, so this case is ~90% likely malignant.  3. The models reported precision (PPV) of 95% indicates that each new positive prediction has a 95% chance of being malignant.  4. The models probability of cancer exceeded a pre-defined threshold |
| 15 | How should AI tools be integrated into existing quality assurance programs in radiotherapy? | 1. AI QA only needs to be performed when the software is updated with a new version of the model.  2. Standard equipment QA procedures are sufficient for AI applications, if the equipment functions as expected the AI model will perform consistently.  3. AI tools should have dedicated QA procedures that monitor both technical performance human factors, and clinical outcomes  4. AI tools don't require QA since they are computer-based, the computer can be programmed with internal checks and tests to ensure safety. |
| 16 | Which change in clinical practice would most likely reduce the accuracy of a static AI model trained to predict patient mortality in radiotherapy patients? | 1. Switching to a different radiotherapy treatment machine of the same modality  2. Reducing quality assurance checks for imaging workflows  3. Decreasing the frequency of multidisciplinary team meetings for case discussion and model oversight  4. Discovery of new systemic therapy (not included in model training) which significantly improves patient RT outcomes |
| 17 | When an AI model trained on data from a single institution is deployed at multiple external hospitals, which statement is true? | 1. Differences in patient demographics, equipment, and practices may degrade model performance  2. A model trained using a deep neural network will automatically adjust to perform optimally at each external site.  3. A model may not perform well outside the local institutions data if it has not passed regulatory clearance (e.g., CE mark, FDA 510(k)) which guarantees equivalent performance across all hospitals that use the model.  4. If trained and validated on a large and diverse internal dataset, the model will generalise well due to standardization in clinical practices between clinics |
| 18 | What is a primary consequence of bias in the healthcare data used to train an AI model? | 1. Bias has no effect on AI model accuracy for the general population, only underrepresented groups.  2. Bias mainly slows the models convergence during training but leaves its final predictions unaffected if class weights are used appropriately.  3. Bias makes the model more conservative, so any errors are spread evenly across all patient populations.  4. Bias can introduce systematic errors that disproportionately harm under-represented or mis-represented patient groups. |
| 19 | In the context of clinical AI models, what does generalisability refer to? | 1. The model performs multiple distinct clinical tasks (e.g., contouring, dose prediction, toxicity forecasting), showing broad clinical knowledge.  2. The models predictions remain stable when its parameters are slightly adjusted during development and training.  3. The model maintains comparable predictive performance on data from different sites, scanners, protocols, and patient subgroups than those used for training.  4. The model maintains comparable predictive performance on the hold-out test dataset that wasn’t used in the training process. |
| 20 | Before first clinical use, which single local activity is the most critical validation step for an AI segmentation model? | 1. Passing a phantom/synthetic dataset test demonstrating geometric accuracy within vendor tolerances.  2. Completing mandatory user training and competency sign-off for all end users.  3. Replicating vendor-reported accuracy/performance scores using the vendors recommended public benchmark locally.  4. Demonstrating acceptable performance on a representative sample of your local patient population |
| 21 | What information would be most important in an AI model's documentation (model card) for clinical end-users informed use? | 1. Comprehensive details about training data characteristics, intended population and known limitations.  2. Technical specifications of the model and how many data points were used for training  3. The physical location and specifications of the servers running the AI model  4. Comprehensive details about the model architecture, validation methodology and performance metrics during validation and testing |
| 22 | What is the most accurate description of how foundational AI models differ from traditional AI models? | 1. Foundation models are dynamic, learning over time based on user input to improve performance to be closer to local practice and requirements.  2. Foundation models are simply larger versions of traditional models that have been trained on more data  3. Foundation models are pre-trained on broad, diverse datasets to learn general representations that can be adapted to multiple downstream tasks  4. Foundation models are specialized AI systems designed exclusively for a single clinical task like organ segmentation |
| 23 | In the context of training an AI model, what is the main purpose of dividing data into training, cross-validation, and test sets? | 1. To train the model on one portion of the data, use another portion to tune model parameters and monitor overfitting, and reserve the final portion for unbiased evaluation of model performance  2. To ensure that the model is exposed to as much data as possible during training for optimal learning and performance reporting  3. To split the data so that the model can learn, self-correct, and then generalize by combining all sets during final prediction  4. To sequentially improve the model's accuracy by retraining on the validation set and finally optimizing on the test set |
| 24 | What is the primary purpose of a loss function during AI model training? | 1. To quantify the proportion of patients that are outside the models training domain  2. To calculate the financial cost of computational resources used during training  3. To quantify the difference between model predictions and ground truth, guiding optimization toward better performance  4. To quantify the final performance of a models prediction vs. ground truth after training the model. |
| 25 | What does "overfitting" mean in the context of AI model development? | 1. When a model architecture is too simple to capture complex anatomical relationships, resulting in poor performance on new patients.  2. When a model performs too well on test data due to data leakage, resulting in poor performance on new patients.  3. When a model processes data faster than the clinical workflow can accommodate  4. When a model memorizes training examples too specifically, resulting in poor performance on new patients |
| 26 | Why is data normalization critical when training AI models on medical imaging data? | 1. To ensure consistent data scales prevent certain features from dominating the learning process.  2. To remove extreme outliers from the dataset to ensure data represents an average patient  3. To group similar patients together in the training process to ensure similar features are clear during the learning process.  4. To randomly shuffle pixel values and increase dataset diversity to prevent certain features from dominating the learning process |
| 27 | What is the primary benefit of data augmentation during AI model training for medical imaging? | 1. To impute (fill in the missing gaps) in the data used for training the model to improve a models performance.  2. To increase available server storage capacity for larger datasets  3. To split the training dataset into smaller more efficient datasets to reduce model computational requirements for lower specification machines.  4. To artificially expand training diversity, improving model robustness to variations in real clinical data |
| 28 | What is the primary advantage of using transfer learning when developing AI models for radiotherapy applications? | 1. Models pre-trained on large datasets can be adapted to specific clinical tasks with limited data  2. Transfer learning automatically ensures compliance with all medical device regulations  3. Pre-trained models will learn and adapt to in-house data once deployed  4. Transfer learning eliminates the need for any local validation or testing by undertaking a fine-tuning training cycle on in-house data |
| 29 | What is a potential benefit of using ensemble methods for AI? | 1. Ensemble methods use a selection of models and picks the best model to for each use case to improve overall accuracy and reliability  2. Using ensemble methods allows multiple datasets to be processed together at the same time for efficiency  3. Ensemble models combine predictions from multiple models to improve overall accuracy and reliability  4. Ensemble methods always produce faster results than single models |

## Supplementary Material B: Item discrimination analysis

*Table S2.* **Item discrimination analysis.** Discrimination index scores and the percentage of correct responses for each of the 22 knowledge items. The discrimination index (calculated using the Kelley 27% method) reflects the ability of an item to distinguish between high- and low-performing respondents.


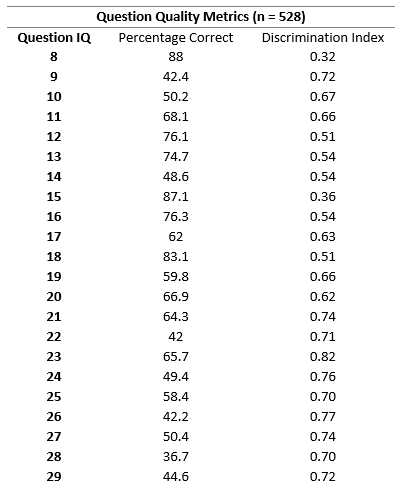


## Supplementary Material C: Bayesian independent analysis


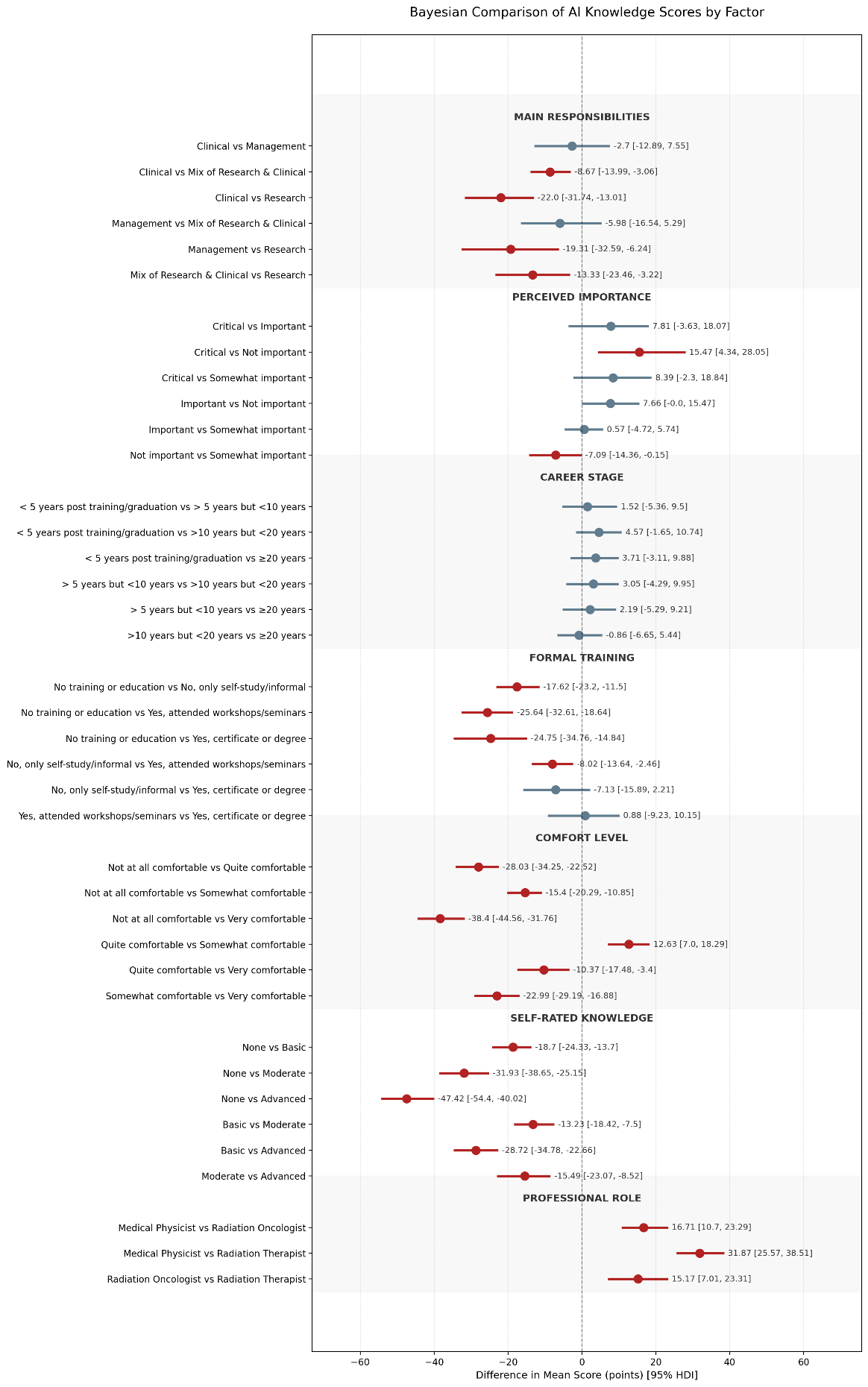


*Figure S1* **Bayesian forest plot of pairwise differences in total AI knowledge scores***.* Points represent the estimated mean difference in score (percentage points) between groups; horizontal bars denote the 95% Highest Density Interval (HDI). Red intervals indicate statistically credible differences where the HDI excludes zero; blue intervals indicate differences where zero falls within the credible range. Estimates were derived using a robust Bayesian Student-t model to minimize the influence of outliers.

Table S3.  **Full Bayesian analysis of pairwise differences.** Detailed output of the robust Bayesian Student-t model comparing total AI knowledge scores between groups. The table reports the mean likely difference (percentage points), the 95% Highest Density Interval (HDI), and the probability of direction. A difference is considered significant if the HDI excludes zero.

| **Factor** |  | **Group1** | **Group2** | **Mean_Diff** | **HDI_2.5%** | **HDI_97.5%** | **Prob_Direction_%** | **Significant** |
| --- | --- | --- | --- | --- | --- | --- | --- | --- |
| professional_role |  | Medical Physicist | Radiation Oncologist | 16.71 | 10.7 | 23.29 | 100 | TRUE |
|  |  | Medical Physicist | Radiation Therapist | 31.87 | 25.57 | 38.51 | 100 | TRUE |
|  |  | Radiation Oncologist | Radiation Therapist | 15.17 | 7.01 | 23.31 | 100 | TRUE |
| knowledge_short |  | None | Basic | -18.7 | -24.33 | -13.7 | 100 | TRUE |
|  |  | None | Moderate | -31.93 | -38.65 | -25.15 | 100 | TRUE |
|  |  | None | Advanced | -47.42 | -54.4 | -40.02 | 100 | TRUE |
|  |  | Basic | Moderate | -13.23 | -18.42 | -7.5 | 100 | TRUE |
|  |  | Basic | Advanced | -28.72 | -34.78 | -22.66 | 100 | TRUE |
|  |  | Moderate | Advanced | -15.49 | -23.07 | -8.52 | 100 | TRUE |
| comfort_level |  | Not at all comfortable | Quite comfortable | -28.03 | -34.25 | -22.52 | 100 | TRUE |
|  |  | Not at all comfortable | Somewhat comfortable | -15.4 | -20.29 | -10.85 | 100 | TRUE |
|  |  | Not at all comfortable | Very comfortable | -38.4 | -44.56 | -31.76 | 100 | TRUE |
|  |  | Quite comfortable | Somewhat comfortable | 12.63 | 7 | 18.29 | 100 | TRUE |
|  |  | Quite comfortable | Very comfortable | -10.37 | -17.48 | -3.4 | 99.7 | TRUE |
|  |  | Somewhat comfortable | Very comfortable | -22.99 | -29.19 | -16.88 | 100 | TRUE |
| formal_training |  | No training or education | No, only self-study/informal | -17.62 | -23.2 | -11.5 | 100 | TRUE |
|  |  | No training or education | Yes, attended workshops/seminars | -25.64 | -32.61 | -18.64 | 100 | TRUE |
|  |  | No training or education | Yes, certificate or degree | -24.75 | -34.76 | -14.84 | 100 | TRUE |
|  |  | No, only self-study/informal | Yes, attended workshops/seminars | -8.02 | -13.64 | -2.46 | 99.8 | TRUE |
|  |  | No, only self-study/informal | Yes, certificate or degree | -7.13 | -15.89 | 2.21 | 94 | FALSE |
|  |  | Yes, attended workshops/seminars | Yes, certificate or degree | 0.88 | -9.23 | 10.15 | 56.6 | FALSE |
| career_stage |  | < 5 years post training/graduation | > 5 years but <10 years | 1.52 | -5.36 | 9.5 | 65.3 | FALSE |
|  |  | < 5 years post training/graduation | >10 years but <20 years | 4.57 | -1.65 | 10.74 | 93.2 | FALSE |
|  |  | < 5 years post training/graduation | >20 years | 3.71 | -3.11 | 9.88 | 86.8 | FALSE |
|  |  | > 5 years but <10 years | >10 years but <20 years | 3.05 | -4.29 | 9.95 | 80.1 | FALSE |
|  |  | > 5 years but <10 years | >20 years | 2.19 | -5.29 | 9.21 | 72.4 | FALSE |
|  |  | >10 years but <20 years | >20 years | -0.86 | -6.65 | 5.44 | 60.5 | FALSE |
| importance_level |  | Critical | Important | 7.81 | -3.63 | 18.07 | 92.2 | FALSE |
|  |  | Critical | Not important | 15.47 | 4.34 | 28.05 | 99.4 | TRUE |
|  |  | Critical | Somewhat important | 8.39 | -2.3 | 18.84 | 94.2 | FALSE |
|  |  | Important | Not important | 7.66 | 0 | 15.47 | 97.4 | FALSE |
|  |  | Important | Somewhat important | 0.57 | -4.72 | 5.74 | 58.5 | FALSE |
|  |  | Not important | Somewhat important | -7.09 | -14.36 | -0.15 | 97.4 | TRUE |
| main_responsibilities |  | Clinical | Management | -2.7 | -12.89 | 7.55 | 69.4 | FALSE |
|  |  | Clinical | Mix of Research & Clinical | -8.67 | -13.99 | -3.06 | 99.9 | TRUE |
|  |  | Clinical | Research | -22 | -31.74 | -13.01 | 100 | TRUE |
|  |  | Management | Mix of Research & Clinical | -5.98 | -16.54 | 5.29 | 85.6 | FALSE |
|  |  | Management | Research | -19.31 | -32.59 | -6.24 | 99.6 | TRUE |
|  |  | Mix of Research & Clinical | Research | -13.33 | -23.46 | -3.22 | 99.4 | TRUE |

# Supplementary Material D: Sensitivity Analysis

To assess whether dropout biased the primary findings, sensitivity analyses were conducted on the full cohort of all initiated sessions for RO, MP and RTTs (n=685). Per-item differences between completers and non-completers were tested using Fisher's exact tests (Holm-corrected within role). Each Table 1 demographic factor was re-tested in three additional cohort definitions (e.g. sessions answering ≥10 items, ≥15 items, and a common-item subset (items answered by ≥75% of initial respondents)) using the same Kruskal-Wallis and pairwise Mann-Whitney framework.

**Drop off Analysis**

| **role** | **n_started Q8** | **n_completed_all 22** | **% completed** | **Median items answered** | **Iqr low** | **Iqr high** | **Median items non-completers only** |
| --- | --- | --- | --- | --- | --- | --- | --- |
| Medical Physicist | 407 | 306 | 75.2 | 22 | 22 | 22 | 5 |
| Radiation Oncologist | 152 | 89 | 58.6 | 22 | 5.75 | 22 | 5 |
| Radiation Therapist | 126 | 80 | 63.5 | 22 | 6 | 22 | 3 |


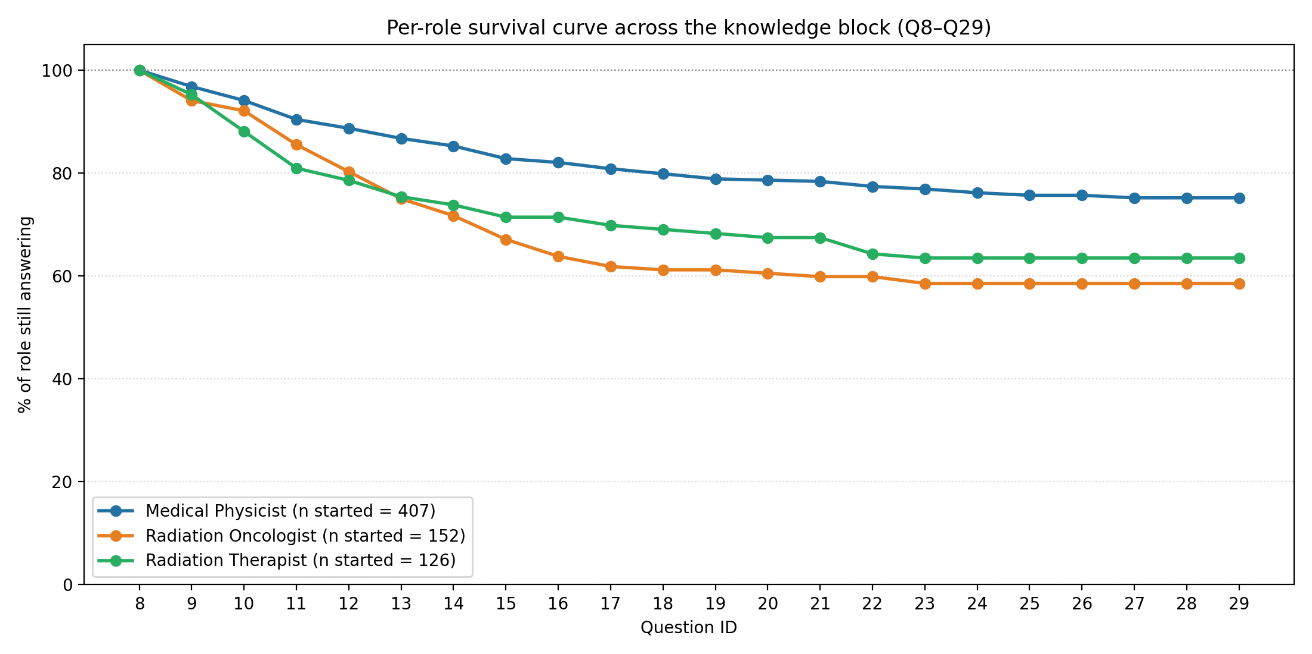


Figure 1. **Per-role survival curve across the knowledge block (Q8–Q29).** Lines show the percentage of respondents from each professional role still actively answering at each question position, among those who initiated the knowledge assessment (MP n=407; RO n=152; RTT n=126). Retention diverged most sharply between Q9 and Q15, after which curves plateaued. Final retention at Q29 was 75% for Medical Physicists, 63% for Radiation Therapists, and 58% for Radiation Oncologists. The early plateau pattern indicates that non-completion was concentrated in respondents who disengaged within the first half of the assessment rather than fatigued mid-block, informing the sensitivity analyses reported

**Per Item completer vs. non-completer**

To address the concern of dropout bias, a per-item sensitivity analysis (Phase 2) confirmed a mild upward selection bias. While we only found one single significant differences on individual items (e.g., the first question for Medical Physicists), the overall trend consistently favoured completers. Across all roles, completers scored a median of 4% to 9% higher on shared items than those who dropped out. This suggests our completer cohort slightly over-represents respondents with higher baseline knowledge. This dynamic strengthens our conclusions in two ways. First, our reported absolute knowledge scores represent a 'best-case scenario' (a ceiling) for workforce readiness, meaning the true clinical need for AI education is likely even greater than reported. Second, our common-subset analysis (Phase 3) demonstrated that the significant knowledge gaps between professions (MP > RO > RTT) are present right from the beginning of the assessment, proving these gaps are fundamental to the professions and not an artifact of later dropout.


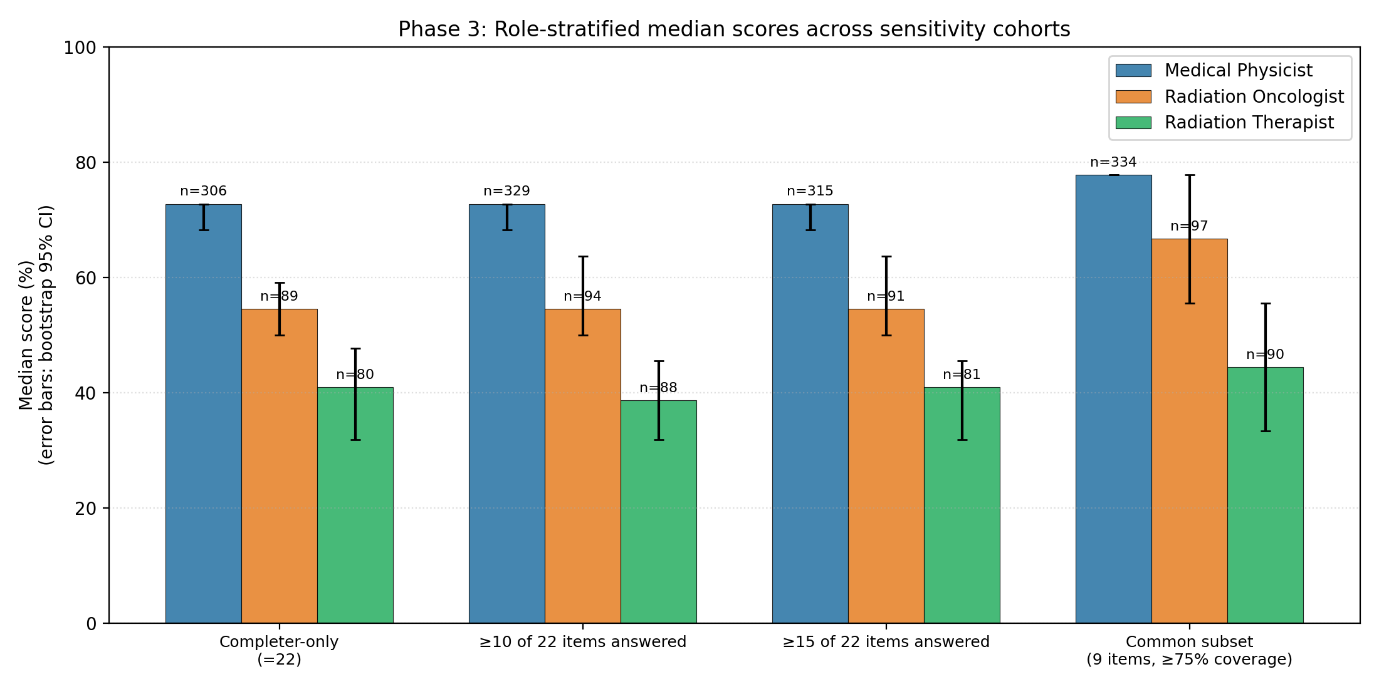


Figure 2 **Median percent-correct knowledge scores for Medical Physicists, Radiation Oncologists, and Radiation Therapists under four cohort definitions**: completer-only (all 22 items, the primary analysis cohort); respondents answering ≥10 of 22 items; respondents answering ≥15 of 22 items; and a common-item subset (9 items answered by ≥75% of role-eligible respondents. The MP > RO > RTT ordering is preserved across all four cohort definitions, with effect sizes (MP–RTT median difference) stable at 32–33 percentage points, indicating that the role finding is robust to dropout-inclusion thresholds. Absolute medians rise modestly in the common-item subset because that subset excludes the more difficult later items.


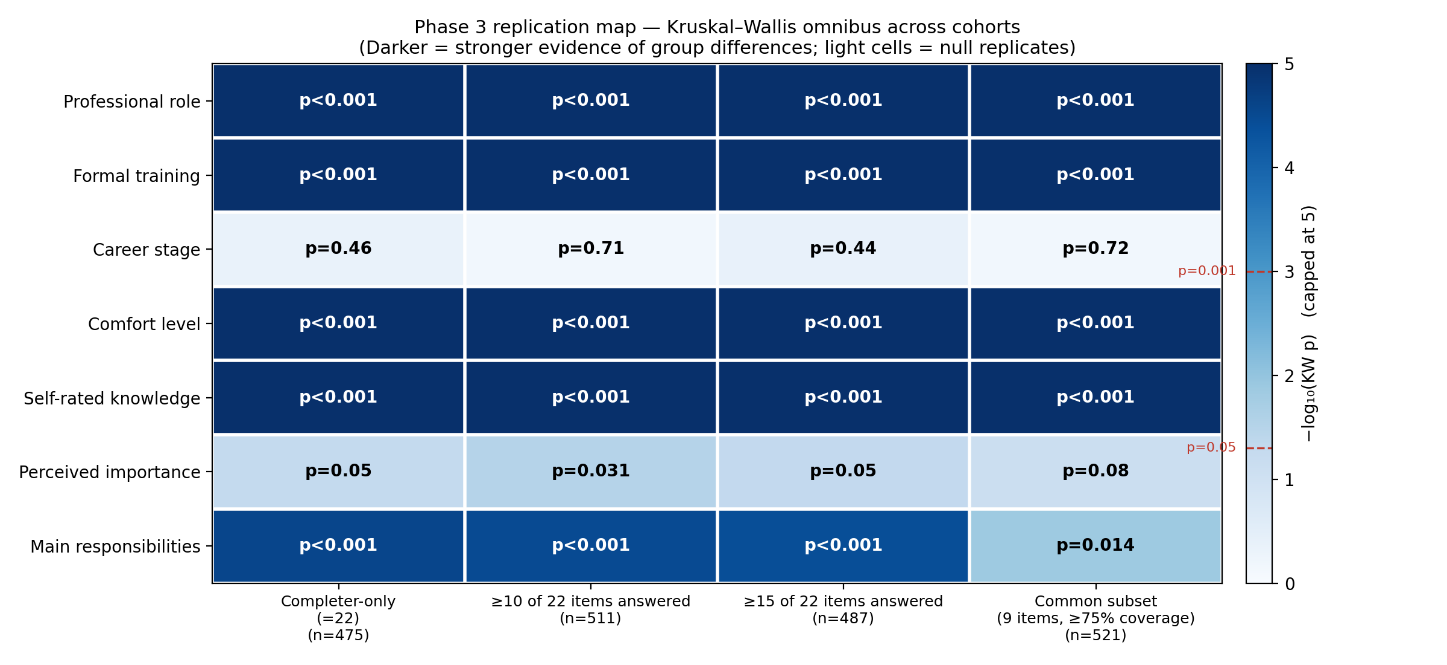


**Figure 3. Replication of Table 1 demographic findings across four sensitivity cohorts**. Heatmap of Kruskal–Wallis omnibus p-values for each of the seven demographic factors from Table 1 (rows) tested in each of four cohort definitions (columns): completer-only (n=475); respondents answering ≥10 of 22 items (n=511); respondents answering ≥15 of 22 items (n=487); and a common-item subset (9 items answered by ≥75% of role-eligible respondents, n=521). As significance p values ranged from 0.72 to <0.001, we scaled cell colour by −log₁₀(p), capped at 5 (darker = stronger evidence of group differences) to make levels of significance clearer; . Dashed red lines on the colour bar mark p=0.05 and p=0.001 thresholds. Five primary findings (professional role, formal training, comfort level, self-rated knowledge, main responsibilities) replicate at p<0.001 across all four cohorts. The null career-stage finding is preserved across all cohorts (p≥0.44). The borderline perceived-importance finding from Table 1 remains borderline across cohorts (p=0.031–0.08). Attenuation of the main-responsibilities finding in the common-subset cohort (p=0.014) reflects reduced power on the 9-item subset rather than a directional
